# The meaning of “coherent” and its quantification in coherent hemodynamics spectroscopy

**DOI:** 10.1142/S1793545818500360

**Published:** 2018-09-27

**Authors:** Angelo Sassaroli, Kristen Tgavalekos, Sergio Fantini

**Affiliations:** Department of Biomedical Engineering, Tufts University, Medford, MA 02155, USA

**Keywords:** Wavelet coherence, phase synchronization, near-infrared spectroscopy, surrogate data

## Abstract

We have recently introduced a new technique, coherent hemodynamics spectroscopy (CHS), which aims at characterizing a specific kind of tissue hemodynamics that feature a high level of covariation with a given physiological quantity. In this study, we carry out a detailed analysis of the significance of coherence and phase synchronization between oscillations of arterial blood pressure (ABP) and total hemoglobin concentration ([Hbt]), measured with near-infrared spectroscopy (NIRS) during a typical protocol for CHS, based on a cyclic thigh cuff occlusion and release. Even though CHS is based on a linear time invariant model between ABP (input) and NIRS measurands (outputs), for practical reasons in a typical CHS protocol, we induce finite “groups” of ABP oscillations, in which each group is characterized by a different frequency. For this reason, ABP (input) and NIRS measurands (output) are not stationary processes, and we have used wavelet coherence and phase synchronization index (PSI), as a metric of coherence and phase synchronization, respectively. PSI was calculated by using both the wavelet cross spectrum and the Hilbert transform. We have also used linear coherence (which requires stationary process) for comparison with wavelet coherence. The method of surrogate data is used to find critical values for the significance of covariation between ABP and [Hbt]. Because we have found similar critical values for wavelet coherence and PSI by using five of the most used methods of surrogate data, we propose to use the data-independent Gaussian random numbers (GRNs), for CHS. By using wavelet coherence and wavelet cross spectrum, and GRNs as surrogate data, we have found the same results for the significance of coherence and phase synchronization between ABP and [Hbt]: on a total set of 20 periods of cuff oscillations, we have found 17 coherent oscillations and 17 phase synchronous oscillations. Phase synchronization assessed with Hilbert transform yielded similar results with 14 phase synchronous oscillations. Linear coherence and wavelet coherence overall yielded similar number of significant values. We discuss possible reasons for this result. Despite the similarity of linear and wavelet coherence, we argue that wavelet coherence is preferable, especially if one wants to use baseline spontaneous oscillations, in which phase locking and coherence between signals might be only temporary.

## 1. Introduction

Recently, our group proposed a novel method, named coherent hemodynamics spectroscopy (CHS), for studying microvascular and microcirculation integrity by inducing controlled hemodynamic changes in living organisms.^1^ These hemodynamic changes may be associated with perturbations in the arterial blood pressure (ABP) that, in human studies, can be induced by a forcing mechanism such as paced breathing,^2,3^ cyclic occlusions and releases of pneumatic thigh cuffs,^4^ head-up tilt protocols,^5^ or squat–stand maneuvers.^6^ For brain studies, the ensuing changes in cerebral blood volume and blood flow can be measured by a number of techniques, including functional magnetic resonance imaging, near-infrared spectroscopy (NIRS), transcranial Doppler ultrasound, and diffuse correlation spectroscopy.^7^ The basic hypothesis of CHS is that tissue with healthy microvasculature and perfusion differs from diseased tissue in terms of the dynamic relationship between blood flow and blood volume (or their covariates such as the tissue concentrations of oxy- [HbO_2_], deoxy- [Hb], and total hemoglobin [Hbt]) in response to hemodynamic perturbations such as those induced by systemic ABP changes. In particular, when perturbations in ABP are the driving force of the observed hemodynamic changes, one can study the phenomenon of cerebral autoregulation (CA), which is critically important for brain health.^7^

The idea of studying the covariation of [Hb] and [HbO_2_] with NIRS to obtain diagnostic or functional information is not new. For example, spontaneous cerebral co-variations of [Hb] and [HbO_2_] were characterized in infants^8,9^ and adults,^10–12^ and the phase relationship of paced-breathing-induced oscillations in cerebral [Hb] and [HbO_2_] was associated with the level of CA.^13^ Furthermore, covarying representations of cerebral [Hb] and [HbO_2_] were proposed for functional brain studies.^14–16^ The novelty of the CHS technique is that it targets a specific kind of cerebral hemodynamics, namely, those that feature a high level of coherence and phase synchronization with a specific driving physiological process. CHS is not only based on the characterization of multi-frequency oscillations or dynamic transients of cerebral [Hb] and [HbO_2_], but it also translates such frequency- or time-resolved dynamic information into physiological quantities on the basis of a CHS mathematical model that treats the cerebral microvasculature as a linear, time-invariant system.^1^ The CHS model shows that oxy- and deoxy-hemoglobin concentrations oscillations, in particular their phase and amplitude relationships, depend on ABP oscillations (and associated blood volume and flow oscillations) through six physiological parameters, including the capillary and venous blood transit times, relative blood volumes in the arterial, capillary, and venous compartments, a measure of CA, etc. These parameters are fitted for in an inversion procedure using our CHS model as a forward solver.^17^ The strengths of our CHS model and its current limitations with respect to others proposed in the literature are described concisely in our previous publications.^7,18^

The CHS treatment of the microvasculature as a linear time-invariant system requires a high level of coherence or covariation between the measured hemoglobin concentration dynamics and the physiological changes of blood volume, blood flow, and oxygen consumption in the investigated tissue. In fact, when a process is stationary, low coherence levels between measured hemoglobin concentrations and underlying physiological processes may reflect a nonlinear relationship, a dependence on additional variables not considered in the CHS model, or contributions from confounds or noise.^19^ This work considers the coherence or covariation between cerebral total hemoglobin concentration [HbT] and systemic ABP, which are directly relevant for CHS, and presents methods to define proper metrics and their critical values for significance.

We note that the study of the significance of metrics for coherence or covariation between signals is a prerequisite for studying CA. For example, in transcranial Doppler, it is required high coherence between ABP and blood flow velocity (BFV) in the middle cerebral artery.^20^ Some NIRS published studies have used ABP and the difference [HbO_2_]-[Hb], which has been considered a surrogate of cerebral blood flow.^21^ The importance of coherence between ABP and cerebral concentrations of oxy- and deoxyhemoglobin was also reported in a transfer function analysis of NIRS signals.^10^ In all of these studies, a coherence greater than a threshold value of about 0.5 was considered to be significant. More generally, a threshold coherence value for significance should result from a statistical test of a null hypothesis of zero coherence, which depends on the degrees of freedom associated with the specific case of data collection and analysis. Following this statistical procedure, lower threshold values in the range 0.1–0.3 have been reported.^19,22,23^ The thresholds of significance for coherence reported in these works were demonstrated by Koopmans^24^ for the case of stationary Gaussian processes and nonoverlapping segments used for the Welch’s periodogram method. A step forward in coherence estimation was achieved by Gallet and Juliene,^25^ in which the authors derived a formula valid also for overlapping segments according to Welch’s method. We also point out the work of Faes *et al.*,^26^ in which the authors used surrogate data for defining a threshold of significance of coherence in cardiovascular variability analysis and showed that such a threshold could be frequency-dependent.

In this work, we find critical values of significance for coherence and phase synchronization of ABP and [HbT] under typical conditions of CHS measurements, and we discuss their relevance for CHS. We have used [Hbt] instead of [HbO] and [Hb], because usually it is the most robust NIRS measurand. However, the results of this study can be applied also for the covariation between [HbO] and ABP, and [Hb] and ABP. These two NIRS measurands would be used in a CA study. A key assumption of the CHS technique and model is that the dynamics of the concentrations of hemoglobin species be mainly driven by a single physiological or functional process, say changes in ABP, in which case they are expected to feature a high level of coherence and covariation with such process. In reality, in addition to ABP, there are a number of factors that can affect the concentrations of oxy- and deoxyhemoglobin in brain tissue: for example, fluctuations in blood CO_2_ levels, changes in oxygen consumption during brain activation, local vasomotion, spontaneous changes in respiratory and heart rates (which do lead to associated changes in blood pressure), Mayer waves, etc. Therefore, in order to minimize the effects of other confounds, we have proposed a protocol for CHS that uses cyclic thigh cuff occlusion and release maneuvers at multiple frequencies, in which the subjects are resting and not engaged in any mental or physical challenge. This protocol of cyclic inflation and deflation of pneumatic thigh cuffs is known to induce cyclic oscillations in ABP.^4^ However, spontaneous oscillations in ABP may also contribute to the observed dynamics of hemoglobin concentration. In transcranial Doppler studies, in which the autoregulation in the macrocirculation is studied, no distinction is made between fluctuations in BFV that are caused by spontaneous or induced oscillations in ABP (as long as the amplitude of blood pressure oscillations is above a certain threshold; see method of coherent averaging^27^). Similarly, in our study, we have not investigated whether blood pressure oscillations are induced by thigh cuff oscillations (this could be done by using the methods discussed later) but we only have focussed on a metric of coherence or covariation between oscillations in ABP and in cerebral hemoglobin concentration.

As a metric of covariation, one could use the magnitude square coherence (linear coherence) and test whether its value at the frequency of cuff pressure oscillations is above a certain threshold of significance, as discussed in the literature quoted earlier. However, we must assume that the whole process under observation is stationary. In our study, we consider a protocol that involves an initial baseline acquisition for 2 min followed by five 2 min time windows in each of which we induce cuff pressure oscillations at different frequencies in the range 0.045–0.083 Hz. Twenty seconds of baseline separates two consecutive time windows. Because of the nature of our protocol, the time series of blood pressure and total hemoglobin is nonstationary (meaning that they have time-varying frequency components), and therefore we have considered more appropriate the wavelet coherence and the method of analytic signal based on the Hilbert transform to define metrics of covariation between ABP and total hemoglobin oscillations. Wavelet coherence was used to define a metric for the time-frequency-resolved value of coherence between ABP and total hemoglobin concentration. Since the coherence between two signals is affected by the covariation of both amplitude and phase of the signals,^28–30^ we also define a metric only based on phase covariation (or phase synchronization) of the two signals. In fact, as a result of a strong amplitude covariation, the coherence of two signals may be significant even when the phase covariation is not.^29^

In summary, in this work, we have designed an *ad hoc* method, tailored to our CHS protocol, in order to investigate the level of coherence and phase synchronization between ABP and cerebral total hemoglobin concentration. Since our protocol induces nonstationary oscillations of ABP and NIRS measurands, we used two metrics suitable for nonstationary data to determine whether total hemoglobin oscillations were directly associated with ABP oscillations: (a) wavelet coherence and (b)phase synchronization index (PSI) which are suitable for nonstationary data. For PSI, the phase between two signals was defined by using the wavelet cross spectrum and also the method of analytic signal, based on the Hilbert transform. We have also used linear coherence for comparison with wavelet coherence. The thresholds or critical values of significance for these two metrics were defined by the method of surrogate data, which involve the derivation of the distributions of the two metrics under a null hypothesis of zero coherence or zero phase synchronization, respectively. Among surrogate data, we have also tested Gaussian random numbers (GRNs), because of the practicality to derive a threshold for our metrics that is only dependent on the protocol and data processing method, but that is not specific to the particular experimental data set. Random numbers have already been used in the literature as surrogate data for statistical inference.^31^

We observe that published NIRS studies on functional connectivity^32–36^ and autoregulation^37–41^ have used the concept of coherence and/or phase synchronization between signals, by using continuous wavelet transform, wavelet coherence, and the analytic signal methods. In a few studies,^32–34,36,37,41^ a method of surrogate data was used in order to assess the significance of the signals covariation. Other NIRS studies in which the method of surrogate data was used for statistical inference are: Bernjak *et al.*,^42^ in which blood flow and oxygen saturation were studied on the skin of human subjects and Bu *et al.*,^43^ in which the authors studied the effect of sleep deprivation. To the best of our knowledge, this is the first NIRS study where the question of covariation between signals has been addressed more critically by using different metrics of covariation and different methods for the estimation of threshold values for significance of those metrics.

## 2. Theory and Methods

### 2.1. Experimental protocol and data acquisition in vivo

The NIRS measurements on human subjects were performed with a commercial frequency-domain NIRS instrument (OxiplexTS, ISS Inc., Champaign, IL, USA). Optical probes connected to the spectrometer delivered light at two wavelengths, 690 nm and 830 nm, at a source–detector distance of 35 mm. The probe was placed against the left side of the subject’s forehead, to access brain tissue in the prefrontal cortex, and secured with a flexible headband. Continuous ABP was recorded with a finger plethysmography monitoring system (NIBP100D, BIOPAC Systems, Inc., Goleta, CA, USA). Pneumatic thigh cuffs were wrapped around both of the subject’s thighs and connected to an automated cuff inflation system (E-20 Rapid Cuff Inflation System, D.E. Hokanson, Inc., Bellevue, WA, USA). The air pressure in the thigh cuffs was continuously monitored with a digital manometer (Series 626 Pressure Transmitter, Dwyer Instruments, Inc., Michigan City, IN, USA). Analog outputs of the ABP monitor and the thigh cuff pressure monitor were fed to auxiliary inputs of the NIRS instrument for concurrent recordings with the NIRS data. All signals were sampled synchronously at an acquisition rate of 12.5 Hz. The protocol consisted of 2 min of baseline acquisition followed by five groups of thigh cuff occlusion and release at the frequencies: 0.0454, 0.0555, 0.0625, 0.0714, and 0.0833 Hz, corresponding to oscillation periods of 22, 18, 16, 14, and 12 s, respectively. Each group consisted of six consecutive cuff inflation/deflation cycles followed by 20 s of rest (where the cuffs stayed deflated). At the end of the last cycle, 1 min of recovery was collected. The order of the cuff frequencies was scrambled in order to have a minimum overlap among the induced frequencies between consecutive groups of oscillations (see Sec. 2.4.2). Specifically, the sequence of frequencies of cuff oscillations was: 0.0625, 0.0833, 0.0454, 0.0714, and 0.0555 Hz. The maximum cuff pressure during the legs occlusion was 200 mmHg. The Tufts University Institutional Review Board approved the experimental protocol, and the subjects provided their informed consent. In this study, we show the results obtained on four subjects.

### 2.2. Wavelet coherence and analytic signal methods

In this section, we describe the wavelet coherence and the analytic signal approach, which we have used in the analysis of our data to obtain measures of coherence (in addition to linear coherence by Fourier transformation) and phase synchronization, respectively. Given a real function *x*(*t*), the continuous wavelet transform *C_x_* (cwt) is defined as:

(1) $$C_{x}(a,b)=\frac{1}{\sqrt{a}}\int_{-\infty }^{+\infty }x(t)\cdot \psi^{*}\left(\frac{t-b}{a}\right)\mathrm{dt}\;=\frac{1}{\sqrt{a}}\left[x(t)⊗\psi^{*}\left(\frac{-t}{a}\right)\right](b),$$

where *φ*(*t*) is the mother wavelet, and *a* and *b* are the scale and location parameters, respectively. In Eq. (1), the * and ⊗ symbols denote the complex conjugate and the convolution operator, respectively.^44^ We can describe intuitively the wavelet coefficients *C_x_*(*a, b*) as measuring the local temporal similarity of the original function *x*(*t*) and a time-scaled version of the original mother wavelet *φ*(*t*). A typical mother wavelet used in cwt is the complex Morlet wavelet, *φ*(*t*):

(2) $$\psi (t)=\frac{1}{\sqrt{\pi f_{b}}}e^{2\pi if_{c}t}e^{-\frac{t^{2}}{f_{b}}},$$

(here we have chosen the form provided in MATLAB documentation: https://www.mathworks.com/help/wavelet/ref/cmorwavf.html) where *f_b_* and *f_c_* are the time decay parameter and the central frequency, respectively. For the actual analysis of data, the variable *t* and the parameter *f_c_* are intended to be normalized to the sampling time and frequency, respectively. Given the mother wavelet, a scaled version of it with scaling parameter *a* is characterized by a characteristic frequency:

(3) $$f=f_{c}∕a.$$

Note that it can be misleading to associate one frequency to a scaled wavelet. In fact, due to its time localization, a wavelet is characterized by a spectrum of frequencies and *f* represents only the value where the maximum of the absolute value of its Fourier transform (FT) is found. Equation (3) is the formula that allows one to shift between scale and characteristic frequency. Time scaling of the mother wavelet is therefore achieved by shrinking (*a* < 1) or dilating (*a* > 1) the wavelet in time, which corresponds to probing a signal locally in a frequency band centered at higher or lower frequencies, respectively.

Given two functions *x*(*t*) and *y*(*t*) the wavelet cross spectrum *C_x,y_* and wavelet coherence Coh_*x,y*_ are defined by:

(4) $$C_{x,y}(a,b)=S(C_{x}^{*}(a,b)C_{y}(\mathrm{ab})),$$

(5) $${\mathrm{Coh}}_{x,y}(a,b)=\frac{{|S[C_{x}^{*}(a,b)C_{y}(a,b)]|}^{2}}{S({|C_{x}^{*}(a,b)|}^{2})S({|C_{y}^{*}(a,b)|}^{2})},$$

where *S* is a smoothing operator in time and scale. Here we have reported the formulas provided in MATLAB documentation (https://www.math-works.com/help/wavelet/ref/wcoherence.html). The time-scale resolved phase difference between the two signals is defined as:

(6) $$\Delta \varphi_{x,y}(a,b)=\varphi_{x}(a,b)-\varphi_{y}(a,b)\;=\mathrm{Arg}(C_{x,y}(a,b)).$$

A different method to define the phase of a time-dependent function is the analytic signal method.^45^ Given a function *x*(*t*), its Hilbert transform (HT) is defined as *H_x_*(*t*):

(7) $$H_{x}(t)=\frac{1}{\pi }p.v.\int_{-\infty }^{+\infty }\frac{x(\tau )}{t-\tau }d\tau \;=p.v.\left[x(\tau )⊗\frac{1}{\pi \tau }\right](t),$$

where p.v. denotes the Cauchy principal value of the integral. One important property of the HT is that it shifts the phase of the Fourier components by ±*π*/2:

(8) $$\tilde{H_{x}(t)}(\omega )=-i\cdot \mathrm{sgn}(\omega )\tilde{x(t)}(\omega ),$$

where the tilde (~) denotes the FT and sgn(*ω*) is the signum function. The analytic signal of a function *x*(*t*) is defined as:

(9) $$z_{x}(t)=x(t)+i\cdot H_{x}(t),$$

and it has the property that its FT contains only positive frequencies. However, the definition of analytic signal by itself does not guarantee a unique definition of instantaneous phase and frequency of a signal, the reason being that the analytic signal may contain a broad spectrum of positive frequencies. The method of analytic signal leads to a unique (noncontradictory) definition of instantaneous phase and frequency of a signal only if the Bedrosian’s product theorem holds true,^45^ which requires that a signal is defined in a “narrow” frequency band. If this requirement is not met, paradoxical results for the instantaneous phase and frequency of a signal are found^46^ (pp. 913–914). Therefore, for a correct definition of instantaneous phase by the analytic signal method, first a narrow band pass filter centered at the frequency of interest is applied to the signal *x*(*t*) and a filtered signal *x_F_*(*t*) is obtained. Afterwards, the analytic signal is defined by:

(10) $$z_{x_{F}}(t)=x_{F}(t)+i\cdot H_{x_{F}}(t),$$

where *H_x_F__* is the Hilbert transform applied to the filtered signal. The instantaneous phase is defined as:

(11) $$\varphi_{x}(t)=\mathrm{Arg}(z_{x_{F}}(t)).$$

Both wavelet transform and the analytic signal method allow one to define an instantaneous phase difference between two signals which are particularly useful under nonstationary conditions. Usually, given a time interval in which the phase difference of two signals is studied, both methods yield a distribution of phase values (one phase value for each time point), and the question arises if the distribution is consistent with the hypothesis of a constant, well-defined phase difference between the signals. If the distribution is peaked around a certain phase value, we could argue in favor of a phase covariation or synchronization of the two signals. On the contrary, if the phase distribution is uniform or rather broad, we conclude that there is no phase covariation or synchronization of the signals. In this study, we have used the concept of PSI which is based on the definition of entropy of a random variable.^47^ Given a discrete random variable with *N* possible values, the definition of Shannon entropy is:

(12) $$E=-\sum_{i=1}^{N}P_{i}\cdot \ln(P_{i}),$$

where *P_i_* is the probability of the *i*th value of the random variable. The PSI is defined as:

(13) $$\mathrm{PSI}=1-\frac{E}{E_{\max}},$$

where *E*_max_ = ln(*N*). Strictly speaking, the Shannon entropy of a random variable is defined by using log_2_, since the maximum entropy coincides with the number of bits necessary to code the maximum information, i.e., when all the values are equiprobable. In general, we can easily demonstrate that, regardless of the base for the logarithm *d* (*d* > 1), the maximum entropy occurs when all the outcomes of the random variable are equiprobable and it coincides with log_*d*_(*N*) On the contrary, the entropy of a random variable is zero only when one outcome is certain (i.e., *P_i_* = 1; *P_j_* = 0, *j* ≠ *i*). From the definition of entropy, it follows that the PSI can take values in the range 0–1 and, specifically, PSI = 1 when *E* = 0, and PSI = 0 when *E* = *E*_max_. Other metrics for phase synchronization are also found in the literature. We mention a metric inversely related to the standard deviation of an angular variable,^44,48^ an index based on conditional probability,^47^ and an index based on mutual information.^49^ We note that different metrics may yield different synchronization values because they are sensitive to different features of the phase distribution of the two signals. For example, the PSI of the relative phase of two signals can have a high value also for a multimodal distribution, i.e., having distinct narrow peaks. However, in this case, the distribution might have a large standard deviation and therefore the related synchronization index might not be significant. More complex measures of synchronization have been considered in the field of electroencephalography (EEG).^50^

### 2.3. Method of surrogate data

We used surrogate data to estimate the statistical distribution that our experimental data would follow under conditions of no coherence or no phase synchronization. The method of surrogate data was initially proposed for assessing conditions of nonlinear dynamics in both univariate and multivariate time series.^49,51–57^ It has also been used for testing significance for linear coherence^26,58^ and phase synchronization of signals.^49,59–62^ Surrogate data are simulated data that are generated under a null hypothesis of zero coherence or zero synchronization between two signals. There are different ways to generate pairs of surrogate data having zero coherence or zero phase synchronization. For example, we could assume that the surrogate data are derived from two independent white noise processes, or instead that they are derived from two independent linear Gaussian processes. Therefore, the null hypothesis of zero coherence or zero phase synchronization will be more specifically linked to the underlying stochastic processes the data are generated from. The method to generate surrogate data from different processes is based on retaining different features of the experimental signals, but in all the cases the temporal structure and/or synchronization present in these signals is destroyed by following a process of randomization. The process of randomization is repeated, and the metrics (coherence and PSI in this study) are recalculated for each pair of surrogate data to generate a distribution of the metrics under the null hypothesis. Afterwards, the values of coherence and PSI obtained for the actual experimental signals are compared with a threshold value derived from the distribution obtained under the null hypothesis (usually the 95th percentile, which corresponds to a typical error of type I, *α* of 0.05).

Some of the most common surrogate data methods are the following, where we used the nomenclature of Paluš.^49^

(1) IID1 surrogates are realization of independent identically distributed (IID) white noise stochastic processes which are obtained by scrambling the order of the original signals with two independent permutations for the samples of each pair of surrogate data. The null hypothesis presented by the IID1 surrogates is that the original signals result from independent white noise processes, so that any temporal structure (and synchronization) present in the original signal is destroyed, and only the distribution of the values is preserved.

(2) IID2 surrogates are obtained by using the same permutation to scramble the order of the original signals. In this case, the null hypothesis is that the original signals are mutually dependent white noise processes, where the distribution of the values and the Pearson correlation coefficient between the original signals are preserved during the process of randomization.

(3) FT1 surrogates are obtained by randomizing the phase of the FT of the signals. The two samples in a pair of surrogate data are obtained by using two independent random sequences for the phase of their FTs. In this case, the spectra (or the autocorrelation functions) of the signals are preserved, and the surrogate data realize the null hypothesis of two independent linear Gaussian stochastic processes that asynchronously oscillate at the same frequencies of the original signals.^49^

(4) FT2 surrogates are obtained in a similar manner as FT1 surrogates but the phases are randomized by using the same sequence in the samples of a pair of surrogate data. In this case, each randomization preserves not only the auto spectra but also the cross spectra. The null hypothesis in this case is that of two linear stochastic processes showing a linear synchronization, which is usually weaker than that from nonlinear stochastic processes.^49^

(5) AAFT1 (amplitude-adjusted FT) surrogate data preserve both the auto spectra and the distribution of values of the signals (which is not preserved by FT1). The null hypothesis in this case is that the signals are a monotonic (static) nonlinear transformation of linear Gaussian processes that oscillate asynchronously at the same frequencies of the original signals.^51^

(6) Similar to FT2, one can define AAFT2, which preserves auto and cross spectra, and distribution of the signals.

(7) GRN (Gaussian random numbers) surrogate data are a sequence of independent Gaussian random numbers. In this case, no feature of the original signals is preserved and these surrogate data present the null hypothesis that the signals result from independent Gaussian white noise processes. Several of these surrogate data were discussed in the work of Paluš,^49^ others like AAFT1 are discussed in the work of Theiler *et al.*,^51^ whereas GRN was adopted in the work of Xu *et al.*^31^ The method AAFT2, to the best of our knowledge, was not discussed previously. Because FT2 (and AAFT2) preserves the cross spectrum of the original data, it was argued that these surrogate data preserve some degrees of linear synchronization and that the rejection of FT2 implies the detection of nonlinearity in the abp *T* phase synchronization.^49^ In this work, we have not investigated this point (i.e., if the type of synchronization between abp and *T* is linear or nonlinear); therefore, we have applied the IID1, IID2, FT1, AAFT1, and GRN surrogate data methods.

### 2.4. Data processing

#### 2.4.1. Optical data and ABP

Oxy- and deoxyhemoglobin concentration changes were calculated from the measured optical intensity changes at two wavelengths (690 nm and 830 nm) by the modified Beer–Lambert law^63,64^ with differential pathlength factors (DPFs) of 7.8 at 690 nm and 7.1 at 830 nm. These values are within a range of values that can be found in the literature for brain tissue. Even though the real DPFs may be different from the ones we assumed, the results of this study (about the significance of interdependence between two signals) are not expected to be significantly affected. The changes of intensity were calculated with respect to a baseline value which was defined as the average intensity value during baseline (first 2 min of data acquisition). Total hemoglobin concentration changes (*T*) were calculated as the sum of oxy- and deoxyhemoglobin concentration changes. ABP was de-meaned and normalized by its mean value (average value during baseline, ABP_0_). The normalized ABP which will be used for studying the covariation with *T* is identified with lower case letters (abp) and defined as abp(*t*) = [ABP(*t*)-ABP_0_]/ABP_0_.

#### 2.4.2. Wavelet coherence and analytic signal

We used the MATLAB built-in function “*wcoherence”* for the calculation of wavelet coherence and cross spectrum between ABP and *T*. We used standard settings of the function, including the complex Morlet wavelet as mother wavelet (https://www.mathworks.com/help/wavelet/ref/wcoherence.html). The output of the function was the time-frequency resolved *wavelet* coherence and cross spectrum between abp and *T*. More precisely, the coherence was a matrix of real numbers (${\mathrm{Coh}}_{T,\mathrm{abp}}^{\mathrm{WT}}(a,b)$; the superscript WT stands for wavelet transform) in the range [0, 1] with 145 rows and a number of columns equal to the number of time points of the original abp and *T* signals. We remind that the parameter *a* is the scale and *b* is time. Each row corresponded to a different scale, therefore to a different characteristic frequency in the range 0.001457–5.968 Hz. The frequencies are obtained by defining the scales using 12 octaves (this number depends on the length of our data) and 12 voices per octave (by default). More specifically, the scales are defined by $a=2^{\left(i_{o}+\frac{i_{v}}{N_{v}}\right)}$, where in our case *i_o_* = 0, 1, …, 12, *i_v_* = 0, 1, …, 11, and *N_v_* = 12. The characteristic frequencies are derived from the scales by the formula: *f* = *f_c_*/*a* where *f_c_* = 6/(2*π*). *F_s_*/2, where *F_s_* is the sampling frequency. The cross spectrum ($C_{T,\mathrm{abp}}^{\mathrm{WT}}(a,b)$) was a matrix of complex numbers, having the same size as the previous one. From the cross spectrum matrix, it was possible to define the time-frequency resolved phase difference between signals (Eq. (6)). In order to assign a value and an error to the coherence and phase difference between abp and *T* in each group of six thigh cuff oscillations, we used the following procedure:

(a) first we identified a range of frequencies associated with the group of six cuff oscillations. In the assumption of sinusoidal oscillations, one group has the absolute value of the FT with a main lobe defined by the frequencies ($f_{\text{cuff}}-\frac{1}{\Delta t}$, $f_{\text{cuff}}+\frac{1}{\Delta t}$) where *f_cuff_* is the cuff frequency and Δ*t* is the duration of the six oscillations;

(b) we identified the characteristic frequencies (i.e., the scales) defined by the wavelet analysis within the main lobe ($f_{\text{cuff}}-\frac{1}{\Delta t}$, $f_{\text{cuff}}+\frac{1}{\Delta t}$);

(c) we defined two temporal arrays obtained by averaging the phase differences ($\langle \Delta \varphi_{T,\mathrm{abp}}^{\mathrm{WT}}(b)\rangle$) and coherence ($\langle {\mathrm{Coh}}_{T,\mathrm{abp}}^{\mathrm{WT}}(b)\rangle$) across the scales in the main lobe. We used circular statistics for averaging the phase differences^65^;

(d) we identified the time range associated with the beginning and the end of a group of six cuff oscillations, namely (*t_b_*, *t_e_*), and calculated the average value and standard deviation of $\langle \Delta \varphi_{T,\mathrm{abp}}^{\mathrm{WT}}(b)\rangle$ in that time range. Average and standard deviation of phase differences were calculated by using circular statistics,^65^ but linear statistics was used for the standard deviation of $\langle {\mathrm{Coh}}_{T,\mathrm{abp}}^{\mathrm{WT}}(b)\rangle$. Therefore, we obtained $\langle \Delta \varphi_{T,\mathrm{abp}}^{\mathrm{WT}}\rangle \pm \sigma_{\Delta \varphi }^{\mathrm{WT}}$ and $\langle {\mathrm{Coh}}_{T,\mathrm{abp}}^{\mathrm{WT}}\rangle \pm \sigma_{\mathrm{Coh}}^{\mathrm{WT}}$ at each cuff frequency *f*_cuff_. The latter values were to be compared with the corresponding threshold values obtained by the surrogate data method. For the calculation of ${\mathrm{PSI}}_{T,\mathrm{abp}}^{\mathrm{WT}}$ (Eq. (13)), we arranged in a circular histogram a subset of the values $\langle \Delta \varphi_{T,\mathrm{abp}}^{\mathrm{WT}}(b)\rangle$, those defined in the interval (*t_b_, t_e_*), by dividing the 360° angle into 72 angular intervals of 5° and calculating the frequency of occurrence as estimate of *P_i_* (see Eq. (12)) in each interval. ${\mathrm{PSI}}_{T,\mathrm{abp}}^{\mathrm{WT}}$ was calculated according to Eq. (13). Note that the calculation of ${\mathrm{PSI}}_{T,\mathrm{abp}}^{\mathrm{WT}}$ was not associated with an error, since the data are used only one time for the estimation of one value of ${\mathrm{PSI}}_{T,\mathrm{abp}}^{\mathrm{WT}}$.

The analytic signals of abp and *T* were defined by first applying a narrow band pass filter to the original signals. More precisely, we generated the filter’s coefficient by using a Parks McClellan algorithm corresponding to a pass band of 0.01 Hz, a stop band of 0.02 Hz and centered at *f*_cuff_. Note that the stop band had similar width of the main lobe of the cuff frequencies, which was in the range 0.015–0.028 Hz. The ripple in the pass band and the attenuation between the stop band and the pass band were less than 0.05. The coefficients of the filter were used as input of a zero-phase filtering procedure (“*filtfilt*” function in MATLAB). The phase between *T* and abp was defined as: $\mathrm{Arg}(\frac{Z_{T_{F}}(t)}{Z_{{\mathrm{abp}}_{F}}(t)})$ where *Z_T_F__*(*t*) and *Z*_abp_*F*__(*t*) are the analytic signals defined after filtering *T* and abp (Eq. (10)). An average phase difference and standard deviation were defined by using circular statistics in the time range where the cuff oscillations occurred (*t_b_*, *t_e_*), therefore obtaining $\langle \Delta \varphi_{T,\mathrm{apb}}^{\mathrm{HT}}\rangle \pm \sigma_{\Delta \varphi }^{\mathrm{HT}}$, where the superscript HT stands for Hilbert transform. We also calculated ${\mathrm{PSI}}_{T,\mathrm{abp}}^{\mathrm{HT}}$ in a similar manner as for ${\mathrm{PSI}}_{T,\mathrm{abp}}^{\mathrm{WT}}$.

#### 2.4.3. Linear coherence

We computed the linear magnitude squared coherence (function “*mscohere*” in MATLAB) and the cross power spectral density (function “*cpsd*” in MATLAB) as measures of coherence and the angle between *T* and abp, respectively, at different frequencies. Both functions are based on the Welch’s averaged modified periodogram method. Default settings of the functions were used; therefore, the signals were divided into eight sections with 50% overlap, each section was windowed with a Hamming window, and eight modified periodograms were computed and averaged. For linear coherence between *T* and abp, we defined $\langle {\mathrm{Coh}}_{T,\mathrm{apb}}^{\mathrm{FT}}\rangle \pm \sigma_{\mathrm{Coh}}^{\mathrm{FT}}$ (the superscript FT means Fourier transform) where the averages and standard deviations were calculated across the frequencies in the range ($f_{\text{cuff}}-\frac{1}{\Delta t}$, $f_{\text{cuff}}+\frac{1}{\Delta t}$). Similarly, for the phase difference, we defined $\langle \Delta \varphi_{T,\mathrm{abp}}^{\mathrm{FT}}\rangle \pm \sigma_{\Delta \varphi }^{\mathrm{FT}}$ and $\langle {\mathrm{Coh}}_{T,\mathrm{Aapb}}^{\mathrm{FT}}\rangle \pm \sigma_{\mathrm{Coh}}^{\mathrm{FT}}$. Note that in this case time-averaging of phase difference and coherence is not possible because the method does not produce time-resolved values. We also note that Fourier methods applied to nonstationary signals often produce results that are not easily interpretable, the reason being that the basis of Fourier decomposition of a signal is sinusoidal function defined at all time points. Nevertheless, since Fourier methods are standard and easier to apply, we have also compared its results with those obtained with the method described earlier. A final note about the phase difference between abp and *T*: with all the methods used in this work (wavelet, Hilbert, and FTs), we report the phase of total hemoglobin with respect to the phase of ABP: 〈Δ*φ*_*T*,abp_〉 = 〈*φ_T_* − *φ*_abp_〉.

#### 2.4.4. Calculating threshold values by the surrogate data methods

Given a surrogate data method (described in Sec. 2.3), we calculated a threshold value of significance for ${\mathrm{PSI}}_{T,\mathrm{abp}}^{\mathrm{WT}}$ and ${\mathrm{PSI}}_{T,\mathrm{abp}}^{\mathrm{HT}}$ by using the following procedure. We generated 100 pairs of surrogate data and for each pair we calculated ${\mathrm{PSI}}_{T,\mathrm{abp}}^{\mathrm{WT}}$ and ${\mathrm{PSI}}_{T,\mathrm{abp}}^{\mathrm{HT}}$. For the calculation of the PSI between each pair of surrogate data, we applied the same procedure used for the calculation of PSI between *T* and abp. These 100 values were arranged in a histogram (distribution) where the range [0, 1] was divided into 50 intervals. The threshold values for the wavelet (${\mathrm{PSI}}_{\mathrm{th}}^{\mathrm{WT}}$) and Hilbert transform (${\mathrm{PSI}}_{\mathrm{th}}^{\mathrm{HT}}$) were defined by those values for which the error type I was less than 5% (i.e., the probability of finding a value higher than the threshold was less than 5% under the null hypothesis). We repeated this procedure three times, and we defined $\langle {\mathrm{PSI}}_{\mathrm{th}}^{\mathrm{WT}}\rangle \pm \sigma_{\mathrm{PSI}}^{\mathrm{WT}}$ and $\langle {\mathrm{PSI}}_{\mathrm{th}}^{\mathrm{HT}}\rangle \pm \sigma_{\mathrm{PSI}}^{\mathrm{HT}}$ calculated as average and standard deviation of the values found in the three repetitions. In a similar manner, we calculated the threshold for wavelet coherence and linear coherence, but in this case the threshold values were calculated only one time by using 100 pairs of surrogate data. We assigned a constant error of 0.05 which corresponded to the partition of the interval [0,1] in 20 sections. A rejection of IID1, IID2, FT1 and AAFT1 (which for almost Gaussian distributions yields results similar to FT1) null hypotheses supports the conclusion that the original signals are phase synchronized.^49^

## 3. Results

### 3.1. Example of raw data and phase difference plots

**Fig. 1.**
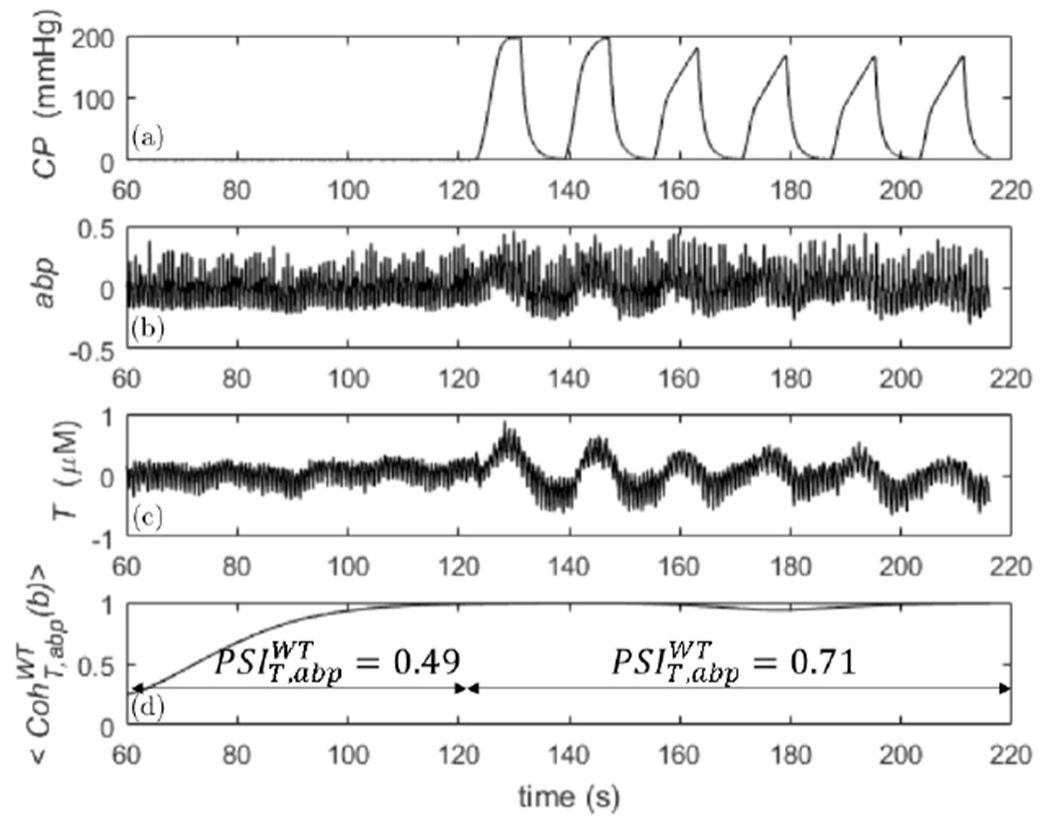
Raw signals and wavelet coherence between abp and *T*, calculated on a subject during the first 2 min of baseline (only last 1 min is shown) and during the first group of six cuff oscillations at *f*_cuff_ = 0.0625 Hz. Panel (a) cuff pressure (CP), panel (b) normalized ABP (abp), panel (c) changes in concentration of total hemoglobin (*T*), and panel (d) wavelet coherence ($\langle {\mathrm{Coh}}_{T,\mathrm{abp}}^{\mathrm{WT}}\rangle$). In panel (d) are also indicated the values of ${\mathrm{PSI}}_{T,\mathrm{abp}}^{\mathrm{WT}}$ during baseline and during the group of six cuff oscillations.

An example of raw data is shown in Fig. 1 (subject 4), which include: cuff pressure (CP; panel (a)), normalized changes in ABP (abp; panel (b)), changes of total hemoglobin concentration (*T*; panel (c)), and wavelet coherence ($\langle {\mathrm{Coh}}_{T,\mathrm{abp}}^{\mathrm{WT}}(b)\rangle$; panel (d)). The plots include the second 1 min of 2 min baseline and the first group of six cuff oscillations at *f*_cuff_ = 0.0625 Hz. In panel (d) are also indicated the values of ${\mathrm{PSI}}_{\mathrm{th}}^{\mathrm{WT}}$ during baseline and during the group of six cuff oscillations.

As it is visible from the raw abp, *T*, and CP signals, the cuff oscillations drive the amplitude and entrainment of abp and *T* oscillations at its own frequency. These oscillations in abp and *T* were not present (or present to a much lesser extent) during baseline. For these reasons, the signals obtained in our protocol are strictly nonstationary, so that nonstationary methods (like wavelet and Hilbert transforms or short time FT (STFT)) are in principle more appropriate. For example, from Fig. 1, we observe how the dynamic entrainment between abp and *T* is captured by both wavelet coherence and PSI: during cuff oscillation, wavelet coherence was significant ($\langle {\mathrm{Coh}}_{T,\mathrm{abp}}^{\mathrm{WT}}\rangle =0.97\pm 0.02$), whereas during baseline it was nonsignificant ($\langle {\mathrm{Coh}}_{T,\mathrm{abp}}^{\mathrm{WT}}\rangle =0.64\pm 0.26$).

**Fig. 2.**
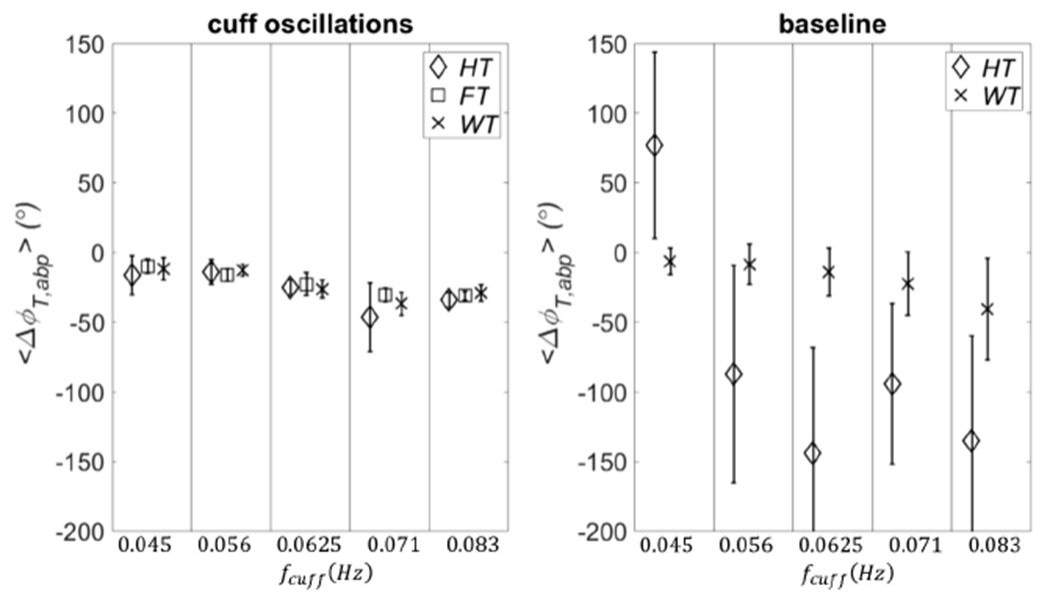
Phase difference between *T* and abp at the cuff frequencies. On the left panel the phase differences were calculated as averages across the main lobe of the cuff frequencies with the analytic signal (HT), wavelet cross spectrum (WT), and cross power spectral density (FT) methods. For the wavelet (WT) and analytic signal (HT) methods, the averages were also carried out in the time intervals when the cuff was oscillating at each specific frequency. On the right panel the time-frequency averages of the phase differences were calculated with analytic signal and wavelet methods during baseline. The vertical lines define the different cuff frequencies. The error bars are the standard deviations across the frequencies of the main lobe of the cuff frequencies (FT) or across the temporal ranges of cuff oscillations (WT and HT).

Similarly, PSI was significant (${\mathrm{PSI}}_{\mathrm{th}}^{\mathrm{WT}}=0.71$) during the cuff oscillations and nonsignificant (${\mathrm{PSI}}_{\mathrm{th}}^{\mathrm{WT}}=0.49$) during baseline. Even linear coherence for this case showed a significant value $\langle {\mathrm{Coh}}_{T,\mathrm{abp}}^{\mathrm{FT}}\rangle =0.90\pm 0.05$. The significance or non-significance of coherence or PSI was true regardless of the null hypothesis for surrogate data. We have found that Fourier, Hilbert, and wavelet-based methods always agreed within errors for the calculation of the phase differences between *T* and abp. One example is shown in Fig. 2 for the same subject of Fig. 1. On the left panel are plotted the average phase differences and standard deviations calculated at the cuff frequencies with the three methods. For the cross power spectral density (FT), these quantities are obtained by considering the frequencies across the main lobe of the cuff frequencies (Sec. 2.4.3), whereas for the analytic signal (HT) and wavelet cross spectrum (WT) methods, after averaging across the frequencies, the final averages and standard deviations were calculated in the time intervals where cuff oscillations occurred (Sec. 2.4.2). On the right panel, the same time-frequency calculation of averages and standard deviations was carried out for the analytic signal method (HT) and wavelet cross spectrum (WT) during baseline. As one can see from these two nonstationary methods, the standard deviations are much smaller during cuff oscillations than during baseline, which can be interpreted as enhanced phase synchronization or entrainment between *T* and abp driven by the cuff. We remind that one of the synchronization indices defined in the literature (not used in this work) is inversely related to the standard deviation of the phase difference distribution.^44,48^ Therefore, the content of Fig. 2 is in agreement with the different values of coherence and PSI found during cuff oscillations and baseline (Fig. 1). Also, from Fig. 2 (right panel), we can see that the phase differences calculated with the analytic signal and wavelet methods do not always coincide (at least when the synchronization of the signals is poor). We will comment on this fact in the discussions. These results confirm that Fourier methods tell us only if abp and *T* are overall coherent or entrained, whereas analytic signal and wavelet methods tell us also when this happens.

### 3.2. Significance of phase synchronization and coherence

**Fig. 3.**
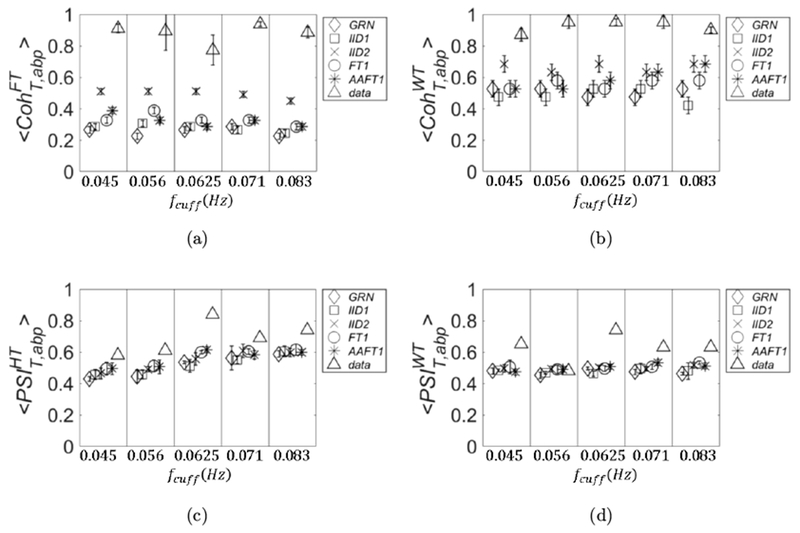
The threshold values of coherence and PSI are shown for five different methods to generate surrogate data together with the values calculated for an experimental data set *in vivo* (subject 1). The *x* axis is defined as in Fig. 2. Specifically, in the four panels are plotted: linear coherence (a), wavelet coherence (b), PSI calculated with analytic signal (c), and PSI calculated with wavelet cross spectrum (d). The methods for surrogate data are: *GRN* (diamonds), IID1 (squares), IID2 (crosses), FT1 (circles), and *AAFT*1 (asterisks). The experimental data are represented by triangles.

A comparison between real data and threshold values for coherence and phase synchronization is presented in Fig. 3 (subject 1). The four panels are organized in the following way: linear coherence $\langle {\mathrm{Coh}}_{T,\mathrm{abp}}^{\mathrm{FT}}\rangle$ (panel (a)); wavelet coherence $\langle {\mathrm{Coh}}_{T,\mathrm{abp}}^{\mathrm{WT}}\rangle$ (panel (b)); PSI calculated with Hilbert transform $\langle {\mathrm{PSI}}_{T,\mathrm{abp}}^{\mathrm{HT}}\rangle$ (panel (c)); and PSI calculated with wavelet transform $\langle {\mathrm{PSI}}_{T,\mathrm{abp}}^{\mathrm{WT}}\rangle$ (panel (d)). In each panel, the *x* axis is defined as in Fig. 2. The threshold values for these parameters were calculated by using five different methods to generate surrogate data: (1) Gaussian random numbers (*GRN*, diamond symbol); (2) independent identically distributed white noise stochastic processes (IID1, square symbol); (3) mutually dependent white noise processes (IID2, cross symbol); (4) linear stochastic processes that asynchronously oscillate at the same frequencies of the original signals (FT1, circle symbol); and (5) monotonic (static) nonlinear transformation of linear Gaussian processes that oscillate asynchronously at the same frequencies of the original signals (AAFT1, asterisk symbol).

The values of these parameters calculated for the experimental data are also shown (data, triangle symbol). We remind that the PSI values for the real data are calculated only one time, and therefore no error is associated to them. In this work, we have not applied statistical tests between experimental data and threshold values; rather, we define an experimental data value as significant if it is not in the error range of the corresponding threshold value. If an experimental data value has an associated error range (as is the case for $\langle {\mathrm{Coh}}_{T,\mathrm{abp}}^{\mathrm{FT}}\rangle$ and $\langle {\mathrm{Coh}}_{T,\mathrm{abp}}^{\mathrm{WT}}\rangle$), we define it as significant if its range does not overlap the range of the threshold value. As we can see from Fig. 3 (subject 1), almost all of the four covariation parameters are significant regardless of the null hypothesis of surrogate data. The only exception is ${\mathrm{PSI}}_{T,\mathrm{abp}}^{\mathrm{WT}}$ (Fig. 3(d)), which is not significant at *f*_cuff_ = 0.056 Hz. However, this value becomes significant if we choose IID1 and GRN as surrogate (we note that for this case the symbol size is larger than the error bars).

**Fig. 4.**
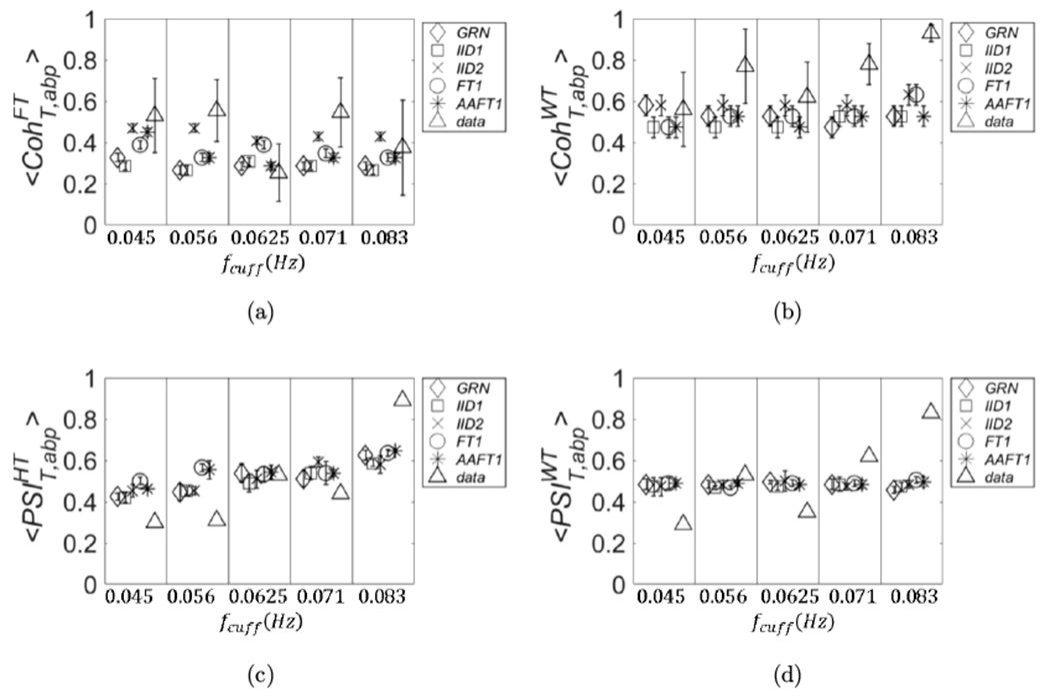
Same as Fig. 3 but for subject 2.

In Fig. 4 (subject 2), we see some discrepancies about the significance of the parameters of covariation. For example, if IID2 is chosen for threshold, $\langle {\mathrm{Coh}}_{T,\mathrm{abp}}^{\mathrm{FT}}\rangle$ (Fig. 4(a)) has no significant values, whereas $\langle {\mathrm{Coh}}_{T,\mathrm{abp}}^{\mathrm{WT}}\rangle$ (Fig. 4(b)) has two significant values (at *f*_cuff_ = 0.071 Hz and 0.083 Hz). ${\mathrm{PSI}}_{T,\mathrm{abp}}^{\mathrm{HT}}$ (Fig. 4(c)) has one significant value (at *f*_cuff_ = 0.083 Hz), whereas ${\mathrm{PSI}}_{T,\mathrm{abp}}^{\mathrm{WT}}$ has three significant values (the same as for $\langle {\mathrm{Coh}}_{T,\mathrm{abp}}^{\mathrm{WT}}\rangle$ and at *f*_cuff_ = 0.056 Hz). If we choose any of the other threshold methods, $\langle {\mathrm{Coh}}_{T,\mathrm{abp}}^{\mathrm{FT}}\rangle$ has two significant values (at *f*_cuff_ = 0.056 Hz and 0.071Hz) and $\langle {\mathrm{Coh}}_{T,\mathrm{abp}}^{\mathrm{WT}}\rangle$ has three (*f*_cuff_ = 0.056 Hz adds to the previous two). The results for PSI do not change. Strictly speaking, if we consider GRN or IID1, $\langle {\mathrm{Coh}}_{T,\mathrm{abp}}^{\mathrm{FT}}\rangle$ has three significant values: the value at *f*_cuff_ = 0.045 Hz adds to the other two significant values found with FT1 and AAFT1.

**Fig. 5.**
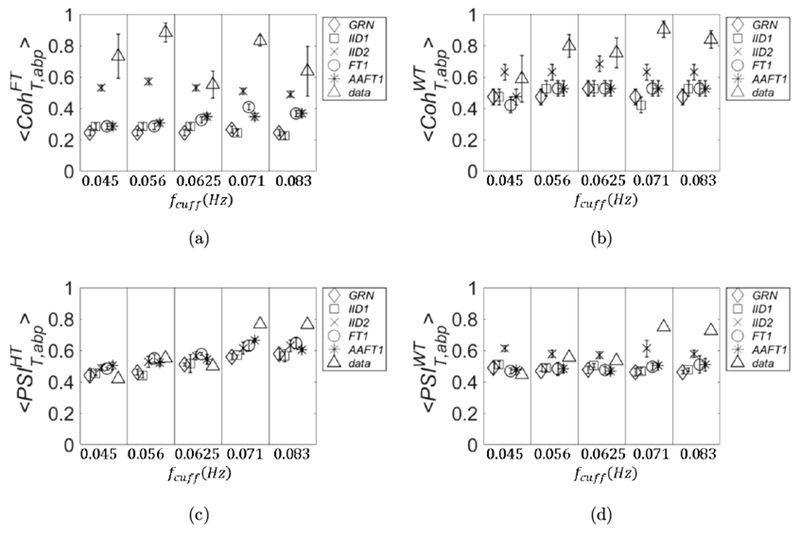
Same as Fig. 3 but for subject 3.

Also in Fig. 5 (subject 3), we see some discrepancies among the parameters of covariation and methods for surrogate data. If we choose IID2 for threshold values, $\langle {\mathrm{Coh}}_{T,\mathrm{abp}}^{\mathrm{FT}}\rangle$ has three significant values (*f*_cuff_ = 0.045, 0.056, and 0.071 Hz); also $\langle {\mathrm{Coh}}_{T,\mathrm{abp}}^{\mathrm{WT}}\rangle$ has three significant values (*f*_cuff_ = 0.056, 0.071, and 0.083 Hz). ${\mathrm{PSI}}_{T,\mathrm{abp}}^{\mathrm{HT}}$ and ${\mathrm{PSI}}_{T,\mathrm{abp}}^{\mathrm{WT}}$ have two significant values (*f*_cuff_ = 0.071 Hz and 0.083 Hz). If we consider any other surrogate data for threshold, all the values are significant for $\langle {\mathrm{Coh}}_{T,\mathrm{abp}}^{\mathrm{FT}}\rangle$; the same is true for $\langle {\mathrm{Coh}}_{T,\mathrm{abp}}^{\mathrm{WT}}\rangle$ with the exception of *f*_cuff_ = 0.045 Hz. For ${\mathrm{PSI}}_{T,\mathrm{abp}}^{\mathrm{HT}}$ another significant value (at *f*_cuff_ = 0.056 Hz) will add to those found with IID2 but only if we choose GRN or IID1 as surrogate data. For ${\mathrm{PSI}}_{T,\mathrm{abp}}^{\mathrm{WT}}$ if we consider any other surrogate data other two significant values (*f*_cuff_ = 0.056 Hz and 0.0625 Hz) will add to those found with IID2.

**Fig. 6.**
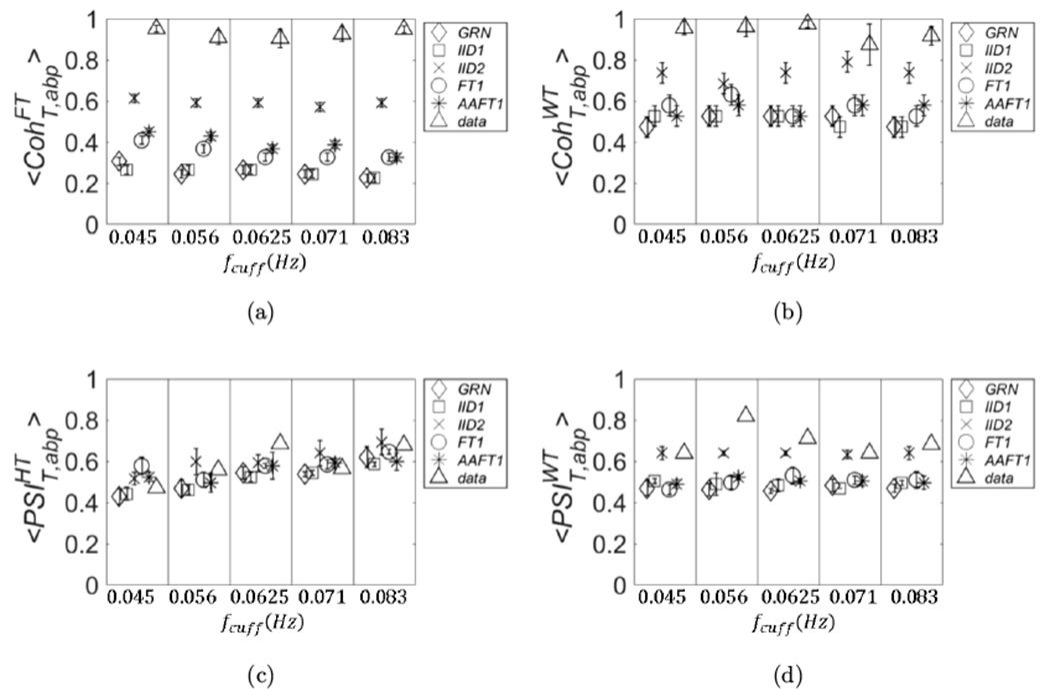
Same as Fig. 3 but for subject 4.

In Fig. 6 (subject 4), we can see that regardless of the surrogate data $\langle {\mathrm{Coh}}_{T,\mathrm{abp}}^{\mathrm{FT}}\rangle$ and $\langle {\mathrm{Coh}}_{T,\mathrm{abp}}^{\mathrm{WT}}\rangle$ show all significant values (the latter with the exception of *f*_cuff_ = 0.071 Hz).

For ${\mathrm{PSI}}_{T,\mathrm{abp}}^{\mathrm{WT}}$ if we choose IID2 we have three significant values (*f*_cuff_ = 0.056, 0.0625, and 0.083 Hz), if we choose any other surrogate data all the values become significant. Different results are obtained for ${\mathrm{PSI}}_{T,\mathrm{abp}}^{\mathrm{HT}}$: if we choose IID2 only one value is significant (*f*_cuff_ = 0.0625 Hz); four more significant values will add if we choose GRN, three if we choose *IID*1, and two if we choose FT1 or AAFT1. The results obtained in this section are summarized in Table 1.

### 3.3. Surrogate data thresholds across subjects

**Fig. 7.**
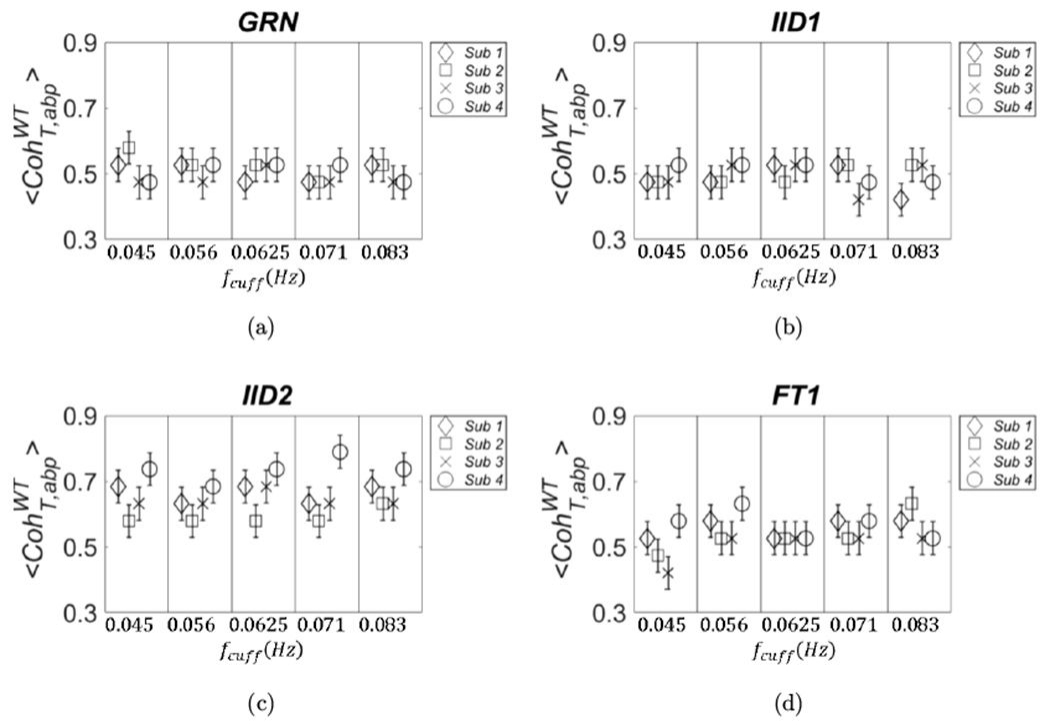
Threshold values obtained from surrogate data for $\langle {\mathrm{Coh}}_{T,\mathrm{abp}}^{\mathrm{WT}}\rangle$ are shown for the four subjects. They refer to GRN (panel (a)), *IID1* (panel (b)), IID2 (panel (c)), and FT1 (panel (d)).

In this section, we show a comparison of the threshold values obtained by different surrogate data across subjects. If possible, one would want to disentangle the threshold values from the particular data set collected on a subject, so that a single threshold value may be used. In this sense, the threshold values obtained with GRN are not data-dependent, but depend only on the details of the protocol and on the details of the calculation method of the metric of covariation. Once those are fixed, the thresholds obtained with GRN are also fixed and could be used in a lookup table of general applicability. All the other surrogate data are obtained by a process of randomization of the experimental data, and the thresholds values derived from them should be recalculated every time new experimental data are collected. Because of the limited statistics used for Figs. 3–6, it is unclear whether the threshold values obtained by different methods for surrogate are subject-dependent. In Fig. 7, the threshold data calculated for $\langle {\mathrm{Coh}}_{T,\mathrm{abp}}^{\mathrm{WT}}\rangle$ are shown for the four subjects. We have omitted only the threshold data calculated with AAFT1 method because of their similarity with those calculated with the FT1 method.

In Fig. 7, we note that the variance across subjects with GRN and IID1 seems to be negligible, but it is not the case for IID2 and FT1. For IID2, the values scale according to the Pearson correlation coefficient between abp and *T*: subject 2 (square symbols in Fig. 7) had the lowest value of 0.33, whereas subject 4 (circle symbols in Fig. 7) had the highest value of 0.59. This discrepancy of the threshold values between subjects obtained with surrogate data IID2 and FT1 is also confirmed by repeating the calculations with higher statistics. We used 1,000 pairs of surrogate data and we extracted ${\mathrm{Coh}}_{T,\mathrm{abp}}^{\mathrm{WT}}$ for each set; this was repeated three times and we computed $\langle {\mathrm{Coh}}_{T,\mathrm{abp}}^{\mathrm{WT}}\rangle \pm \sigma_{\mathrm{Coh}}^{\mathrm{WT}}$. For this calculation, we also chose a more refined partition of the interval (0,1) which was divided into 50 sections.

**Fig. 8.**
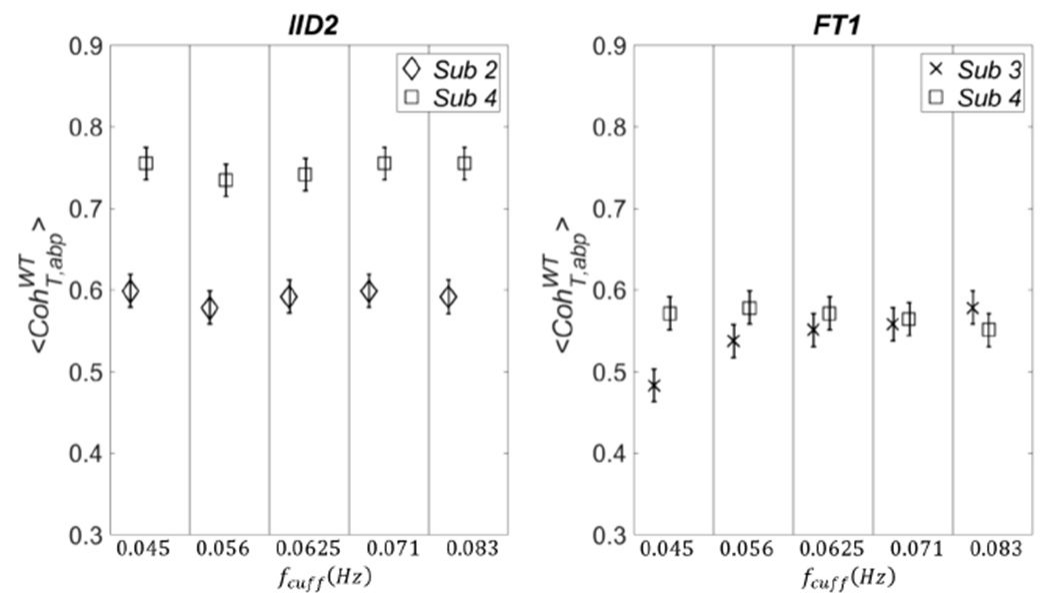
Threshold values obtained from surrogate data for $\langle {\mathrm{Coh}}_{T,\mathrm{abp}}^{\mathrm{WT}}\rangle$ are shown for higher statistics (1,000 pairs of surrogate data) by using IID2 (left panel) and FT1 (right panel).

**Fig. 9.**
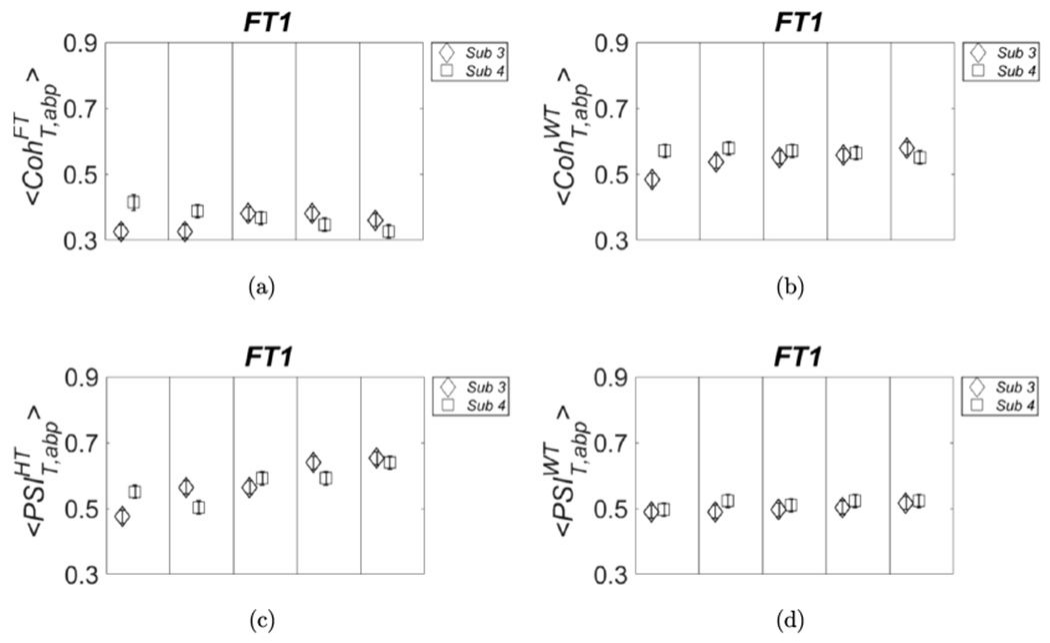
Threshold values obtained with surrogate data FT1 for: $\langle {\mathrm{Coh}}_{T,\mathrm{abp}}^{\mathrm{FT}}\rangle$ (panel (a)), $\langle {\mathrm{Coh}}_{T,\mathrm{abp}}^{\mathrm{WT}}\rangle$ (panel (b)), $\langle {\mathrm{PSI}}_{T,\mathrm{abp}}^{\mathrm{HT}}\rangle$ (panel (c)) and $\langle {\mathrm{PSI}}_{T,\mathrm{abp}}^{\mathrm{WT}}\rangle$ (panel (d)). We used higher statistics as in Fig. 8.

The standard deviation $\sigma_{\mathrm{Coh}}^{\mathrm{WT}}$ is defined as the maximum between the one obtained from the three runs and 0.02 (width of each section). The results are shown in Fig. 8, in which we can see that subject-dependent thresholds can happen at all frequencies (IID2; left panel) or only at specific frequencies (FT1; right panel). In Fig. 9, we show the plots referring to the threshold values calculated with FT1 for the four parameters of covariation: $\langle {\mathrm{Coh}}_{T,\mathrm{abp}}^{\mathrm{FT}}\rangle$ (panel (a)), $\langle {\mathrm{Coh}}_{T,\mathrm{abp}}^{\mathrm{WT}}\rangle$ (panel (b)), $\langle {\mathrm{PSI}}_{T,\mathrm{abp}}^{\mathrm{HT}}\rangle$ (panel (c)), and $\langle {\mathrm{PSI}}_{T,\mathrm{abp}}^{\mathrm{WT}}\rangle$ (panel (d)). The results are shown for subject 3 (diamond symbols) and subject 4 (square symbols), and they were calculated by using better statistics (1,000 pairs of surrogate data) as in Fig. 8.

As one can see in Fig. 9, also for other parameters of covariation, the threshold values calculated from surrogate data may depend on the subject; therefore, they should be recalculated for better accuracy when a new data set on a different subject is collected. Finally, for all the four parameters of covariation the widest variance across subjects is found by using IID2 surrogate data (results not shown).

### 3.4. How many frequencies are induced?

Last, we discuss the effectiveness of our protocol to induce *coherent* oscillations of *T* and abp. One direct way (not used in this work) is to assess the coherence/synchronization between abp and cuff signal and *T* and cuff signal separately (at a given *f*_cuff_), by using one of the surrogate data described before. One indirect way is to compare the coherence/synchronization between abp and *T* during cuff oscillations and during baseline. If the coherence/phase synchronization is higher during cuff oscillations than baseline (at a given *f*_cuff_), we say that a particular frequency *f*_cuff_ was induced by the cuff operation. This point can only be studied with nonstationary methods; therefore, we used three of the four parameters of covariations, namely, $\langle {\mathrm{Coh}}_{T,\mathrm{abp}}^{\mathrm{WT}}\rangle$, $\langle {\mathrm{PSI}}_{T,\mathrm{abp}}^{\mathrm{HT}}\rangle$, and $\langle {\mathrm{PSI}}_{T,\mathrm{abp}}^{\mathrm{WT}}\rangle$. These parameters were calculated during the periods of cuff oscillations and also during baseline, and their significance was decided by assuming the threshold values calculated with FT1 surrogate data (Figs. 3–6). The results are summarized in Table 2. The ratio of successfully induced oscillations varies among the three methods: 17/20 ($\langle {\mathrm{Coh}}_{T,\mathrm{abp}}^{\mathrm{WT}}\rangle$), 11/20 ($\langle {\mathrm{PSI}}_{T,\mathrm{abp}}^{\mathrm{HT}}\rangle$), and 16/20 ($\langle {\mathrm{PSI}}_{T,\mathrm{abp}}^{\mathrm{WT}}\rangle$). We can also see that some coherent oscillations were present at baseline in subject 3 (according to $\langle {\mathrm{Coh}}_{T,\mathrm{abp}}^{\mathrm{WT}}\rangle$ and $\langle {\mathrm{PSI}}_{T,\mathrm{abp}}^{\mathrm{WT}}\rangle$) and in subject 4 (according to $\langle {\mathrm{PSI}}_{T,\mathrm{abp}}^{\mathrm{WT}}\rangle$). Similar results can be expected also by using other surrogate data.

## 4. Discussion

In this section, we address some questions arising from the choice of surrogate data and about the significance of the four parameters of covariation considered by us.

In our data analysis, different surrogate data yield comparable threshold values for significant covariation. However, on average, GRN and IID1 yield slightly lower threshold values, followed by FT1 and AAFT1, and last by IID2. Except GRN, this hierarchy of threshold values according to different null hypothesis reflects the information of the original data which is preserved after different processes of randomization. For IID1, only the distribution of the original data is preserved in the surrogate data, but oscillations and any kind of temporal feature are absent. For FT1, the auto spectra of the original data are preserved, and therefore the surrogate data are independent realization of the original data with de-synchronized oscillations (if they were present in the original data). AAFT1 preserves both auto spectra and the distribution of the original data. It is expected that the threshold values calculated with FT1 and AAFT1 are greater than those found with GRN and IID1 and that the difference is related to the broadness of the spectrum of the original signals. In fact, if two signals are narrow band (ideally only one or few Fourier components), the randomization of the Fourier components will not destroy (or destroy only partly) the coherence/synchronization of the signals calculated at the center frequency of the band. On the contrary, GRN is characterized by a constant power spectrum (independent on frequency) where the Fourier components have unrelated phases, therefore yielding lower values of PSI. Therefore, for the case of a narrow band spectrum, the threshold values obtained with FT1 would be progressively larger (as the band width of the spectrum is decreased) than those obtained with GRN. We verified this point by using finite sinusoidal oscillations as test signals (results not shown). Similar results were found by Faes *et al.*^26^ by comparing IID1 and FT1 for studying the threshold of linear coherence in cardiovascular variability analysis (in our study we have found the same thresholds with IID1 and GRN). The authors concluded that the advantage of using FT1 (for controlling precisely error type I) was clear only for narrow band signals. While for broad band signals, the two surrogate data methods could be used interchangeably.

The method IID2 yields threshold values greater than those obtained with FT1 and AAFT1. This result was not found in the work of Paluš^49^; on the contrary, the author found similar threshold values for IID1 and IID2 when studying the synchronization of the number of solar spots and the temperature in Prague, as function of the calendar year. Since in our study ABP and *T* are measured independently, any correlation of the signals cannot be the result of correlated noise, but only due to a true covariation of the signals. Therefore, we argue that the process of randomization that preserves the Pearson correlation coefficient might be too strict and should not be chosen. In fact, in the limit case in which the Pearson coefficient is “1” (one signal is the scaled version of the other), IID2 yields a threshold value of “1”, causing an error type II of 100%.

The practical importance of using GRN as surrogate data is that the threshold values depend only on the details of the protocol and data acquisition (sampling frequency, cuff frequencies, time span of cuff oscillations) and data processing (filter bandwidth for the HT, mother wavelet choice for WT) but not on the particular data collected on a subject. In this study, we have found that GRN yielded results that are similar to the other methods. For example, by looking at Table 1, if we consider $\langle {\mathrm{Coh}}_{T,\mathrm{abp}}^{\mathrm{FT}}\rangle$, the ratio of significant oscillations was 18/20 with GRN and 17/20 with FT1; with $\langle {\mathrm{Coh}}_{T,\mathrm{abp}}^{\mathrm{WT}}\rangle$ both surrogate data yielded a ratio of 17/20 significant oscillations. By using $\langle {\mathrm{PSI}}_{T,\mathrm{abp}}^{\mathrm{HT}}\rangle$ the ratio was 14/20 with GRN and 11/20 with FT1. Finally, if we consider $\langle {\mathrm{PSI}}_{T,\mathrm{abp}}^{\mathrm{WT}}\rangle$ the ratio was 17/20 with GRN and 16/20 with FT1. The reason for comparing GRN and FT1 is because they yield (on average) slightly lower and slightly higher threshold values (Fig. 7), respectively. These results suggest that GRN surrogate data can be used to obtain reliable threshold values of coherence and phase synchronization in a typical protocol used for CHS. We have also carried out PSI calculation by using the MATLAB function “*cwt*” (continuous wavelet transform) by using both Morse wavelet and complex Morlet wavelet (having *f_b_* = 2, *f_c_* = 6/(2*π*)), obtaining comparable threshold values by using different surrogate methods as those found with the function “*wcoherence*”.

Another question we want to address is about the comparison between stationary methods (represented here by $\langle {\mathrm{Coh}}_{T,\mathrm{abp}}^{\mathrm{FT}}\rangle$) and nonstationary methods (represented here by $\langle {\mathrm{Coh}}_{T,\mathrm{abp}}^{\mathrm{WT}}\rangle$, $\langle {\mathrm{PSI}}_{T,\mathrm{abp}}^{\mathrm{HT}}\rangle$, and $\langle {\mathrm{PSI}}_{T,\mathrm{abp}}^{\mathrm{WT}}\rangle$). In particular, it makes sense to compare $\langle {\mathrm{Coh}}_{T,\mathrm{abp}}^{\mathrm{FT}}\rangle$ with $\langle {\mathrm{Coh}}_{T,\mathrm{abp}}^{\mathrm{WT}}\rangle$ because they are both a measure of coherence between two signals. From the significance viewpoint, even though the two metrics did not always agree, they shared a similar number of significantly induced oscillations for the four subjects investigated in this study (Table 1). However, we remind that the meaning of the significance is different for the two metrics: a significant $\langle {\mathrm{Coh}}_{T,\mathrm{abp}}^{\mathrm{FT}}\rangle$ at a frequency *f*_cuff_ means that during the entire experiment the two signals were overall coherent at that frequency; in contrast, a significant $\langle {\mathrm{Coh}}_{T,\mathrm{abp}}^{\mathrm{WT}}\rangle$ at *f*_cuff_ means (at least for this study) that the two signals were coherent during the time period when the cuff was oscillating at the frequency *f*_cuff_ (note that $\langle {\mathrm{Coh}}_{T,\mathrm{abp}}^{\mathrm{WT}}\rangle$ can be studied, in principle, in any time interval). One possible explanation of the similarity of these two metrics is that our particular protocol, due to the wide overlapping of frequency bands induced by the cuff oscillations (at least for some cuff oscillation frequencies), can be considered weakly nonstationary. In fact, by using $\langle {\mathrm{Coh}}_{T,\mathrm{abp}}^{\mathrm{WT}}\rangle$ we have found that a particular frequency *f*_cuff_ can be significant also in a time period when the cuff was oscillating at another (nearby) frequency. Moreover, because $\langle {\mathrm{Coh}}_{T,\mathrm{abp}}^{\mathrm{FT}}\rangle$ is an overall measure of coherence (as if the signals were strictly stationary), it is affected by the coherence during baseline, which is not considered in our significance analysis with $\langle {\mathrm{Coh}}_{T,\mathrm{abp}}^{\mathrm{WT}}\rangle$. For example, by looking at Table 2, it seems that by using $\langle {\mathrm{Coh}}_{T,\mathrm{abp}}^{\mathrm{WT}}\rangle$, the cuff frequencies were all significant also at baseline on subject 3. This observation might explain the higher ratio of significant frequencies found for subject 3 by using $\langle {\mathrm{Coh}}_{T,\mathrm{abp}}^{\mathrm{FT}}\rangle$ compared with those found with $\langle {\mathrm{Coh}}_{T,\mathrm{abp}}^{\mathrm{WT}}\rangle$ (Table 1). Given the relevance of time-resolved measures of coherence, for example, to identify coherent hemodynamics oscillations at rest (when no ABP perturbations are induced), we favor measures of coherence based on the wavelet transform rather than the FT for CHS. However, the nonstationary approach of the STFT is also suitable for CHS.

Another interesting question we want to address is about the relationship between coherence and phase covariation (or synchronization). There is a large amount of literature (especially in the field of EEG and magnetoencephalography (MEG)) pointing out that coherence is affected by both phase synchronization and covariation of amplitudes.^28–30,66^ For example, Srinath and Ray^29^ found that even when the phase relationship between two EEG channels is random, a strong amplitude covariation may introduce a significant coherence. Also, in an MEG study by Tass *et al.*,^47^ the authors found a significant coherence between channels without a detection of significant phase synchronization. These results, observed in two different research fields, point to the conclusion that a significant phase synchronization might be more difficult to achieve than a significant coherence. In our study, we can compare the coherence and phase synchronization metrics by releasing the assumption of stationarity. In fact, if we consider $\langle {\mathrm{Coh}}_{T,\mathrm{abp}}^{\mathrm{WT}}\rangle$ and $\langle {\mathrm{PSI}}_{T,\mathrm{abp}}^{\mathrm{WT}}\rangle$ from Table 1, we can see that these two metrics have a similar significance across the four subjects. In particular, the ratios of significant induced oscillations for $\langle {\mathrm{Coh}}_{T,\mathrm{abp}}^{\mathrm{WT}}\rangle$ and $\langle {\mathrm{PSI}}_{T,\mathrm{abp}}^{\mathrm{WT}}\rangle$ are: 17/20, 17/20 for GRN, same ratios of GRN for IID1, 17/20, 16/20 for FT1 and same ratio of FT1 for AAFT1, respectively. These results point to the conclusion that wavelet coherence is mainly affected by phase synchronization, at least when the phase is calculated with wavelet cross spectrum, so that $\langle {\mathrm{Coh}}_{T,\mathrm{abp}}^{\mathrm{WT}}\rangle$ may be a good option to assess the level of both coherence and phase synchronization in CHS.

We want also to discuss the differences of the results obtained with the two PSI metrics, which differ only for the way the phases are calculated. First, by looking at Figs. 3–6, we notice a qualitative difference between $\langle {\mathrm{PSI}}_{T,\mathrm{abp}}^{\mathrm{HT}}\rangle$ and $\langle {\mathrm{PSI}}_{T,\mathrm{abp}}^{\mathrm{WT}}\rangle$. In fact, for all the subjects and for any surrogate data, the threshold of $\langle {\mathrm{PSI}}_{T,\mathrm{abp}}^{\mathrm{HT}}\rangle$ has a linear trend with *f*_cuff_, whereas $\langle {\mathrm{PSI}}_{T,\mathrm{abp}}^{\mathrm{WT}}\rangle$ is independent of it. These results can be explained if one thinks that a critical parameter for PSI_th_ is the product Δ*f* · Δ*t*, where Δ*f* is the bandwidth of the filter used and Δ*t* is the period of observation (in this study, the operating time of the cuff). More specifically, we can say that PSI_th_ is approximately inversely proportional to Δ*f* · Δ*t*. For the HT method, we have filtered the data by using a Parks McClellan filter with constant band width Δ*f*; therefore, in this case, ${\mathrm{PSI}}_{\mathrm{th}}^{\mathrm{HT}}$ is inversely proportional to Δ*t*. For the protocol, we have induced the same number of oscillations at each value of *f*_cuff_; therefore, the lowest and highest *f*_cuff_ are associated with the lowest and highest ${\mathrm{PSI}}_{\mathrm{th}}^{\mathrm{HT}}$, respectively. By the definition of WT (Eq. (1)), we can see that the WT is a convolution and that at each scale (i.e., each characteristic frequency, *f* = *f_c_*/*a*), the scaled mother wavelet acts as a filter (at the frequencies centered around *f*) to the entire signal. We can prove that for any scaled wavelet of the Morlet mother wavelet Δ*f*/*f* (where Δ*f* is the bandwidth of the scaled wavelet) is a constant. Therefore, for the wavelet “filter” Δ*f* is directly proportional to *f* ≅ *f*_cuff_ and Δ*f* · Δ*t* is constant for different *f*_cuff_. About the significance, if one compares the two PSI metrics across the subjects (Table 1) by using GRN and FT1, we get ratios of significant induced oscillations for $\langle {\mathrm{PSI}}_{T,\mathrm{abp}}^{\mathrm{HT}}\rangle$ and $\langle {\mathrm{PSI}}_{T,\mathrm{abp}}^{\mathrm{WT}}\rangle$ of 14/20 and 17/20 for GRN and 11/20 and 16/20 for FT1, respectively. In the work of Bruns,^30^ the author presented theoretical arguments to infer the similarity among STFT, wavelet transform, and Hilbert transform. Bruns argued that the differences between the three non-stationary methods could be narrowed down to the choice of a window function in the three approaches, and if one chose the same window function there would be no difference among these methods. The author, after choosing similar window functions for STFT, WT, and HT, showed a qualitative comparison of the spectral amplitudes of some EEG data with the three methods. The subject of the similarity of phase synchrony with WT and HT was addressed, at least partly in the work of Le Van Quyen *et al.*^28^ In the work of Le Van Quyen *et al.*, the authors presented a figure (Fig. 7(d)) in which the number of significant synchronous events found with HT and WT was comparable; however, it is not clear whether they applied the same metric or two different metrics to calculate synchronicity of events. In their work, they described the metric of phase locking value (PLV) and single-trial PLV (SPLV) when using wavelet transform. These metrics are related to angular standard deviation of the phase difference between two signals. Instead, they described PSI and mutual information when using Hilbert transform. We have already discussed in the introduction that the metric based on angular standard deviation and that based on PSI may not always agree. In our work, we have chosen the same metric (PSI) for the two ways (based on HT and WT) of calculating the phase difference between two signals. It is possible that the discrepancies found between the significance of the two PSI metrics in our study are due to the different window functions, which in the wavelet approach is the shape of either the real or imaginary part of the wavelet and for the Hilbert transform is the shape of the impulse response function of the band pass filter.

Similar to Bruns’ work,^30^ we want express our ideas about a comparison among STFT, WT, and HT for studying coherence and synchronization in CHS. There are basically two reasons to prefer STFT or WT instead of HT: (1) STFT and WT yield two time–frequency resolved parameters (coherence and phase difference between two signals), whereas HT yields only one (the phase difference); (2) STFT and WT imply less computational effort than HT, at least if we want to determine the instantaneous phase at a number of distinct frequencies, since HT requires previous application of narrow band filtering at each frequency which can be a computationally heavy process. We note though that these drawbacks of HT might be reduced if one applies the empirical mode decomposition^67^ (EMD), previous to the application of the HT to the data in order to find the so-called Hilbert spectrum (where both the amplitudes and the instantaneous frequencies of the intrinsic mode functions (IMF) are expressed as a function of time). However, in principle, one would want to decompose the original signals in IMFs which are associated with clearly identifiable physical processes which still seem to be an issue for EMD.^68^

About the comparison between WT and STFT, research in our group applied to CHS has found that these two methods are similar and could both be used equally well for data analysis. In STFT, given a certain window size and type, it is more straightforward to assign a time and frequency resolution. On the contrary, as we have repeatedly discussed in this work, WT deals with scales and the “translation” of scales into frequencies should be done always with caution. In particular, one should not confuse the characteristic frequencies as the only frequencies of the scaled wavelets and their spacing (Δ*f*) as the frequency resolution associated with WT. For example, in our study, for a typical characteristic frequencies list output by the MATLAB function “*wcoherence*”, the spacing Δ*f* becomes smaller than the inverse of the total experiment duration, which obviously cannot be the true resolution.

In this study, we have focussed on the covariation between two independent data set like abp and *T*. However, in NIRS, we are often interested to study the covariation between oxy- and deoxyhemoglobin concentrations. These hemoglobin species are often derived by using mBLL, therefore, by using a linear combination of the same normalized changes of intensities at two wavelengths. The linear combination introduces a spurious correlation between the two hemoglobin species. For example, when we use λ_1_ = 690 nm and λ_2_ = 830 nm, we can show that even when two sequences of random numbers are chosen as normalized intensities at the two wavelengths, the phase difference between oxy- and deoxyhemoglobin is weakly peaked around 180°. Given a fixed protocol, the study of coherence and PSI in this case shows that higher thresholds are found for oxy-and deoxyhemoglobin than for abp and *T*.

## 5. Conclusions

In this work, we have investigated the significance of covariation between ABP oscillations (abp) and cerebral concentrations of total hemoglobin (*T*) in human subjects during a protocol involving cyclic inflation and release of pneumatic thigh cuffs. We focussed on the question of covariation, in the sense of both coherence and phase synchronization, between abp and *T*, which is directly relevant in the new technique of CHS. Even though the data processing method was tailored to our protocol and CHS, the scope of our work is broader because it aims at defining suitable metrics of significant covariation for any pair of signals.

The main goal of this work was to determine criteria to identify coherent/synchronous hemodynamic oscillations that are suitable for CHS. On the basis of our results, we propose to use independent Gaussian random numbers (*GRN* surrogate data) to generate a null-hypothesis statistical distribution of coherence, whose 95th percentile is taken as a critical (or threshold) value for significance. Here, we have focussed on coherence between abp and *T*, but the methods presented are applicable to the characterization of coherence between any physiological and cerebral hemodynamics measures. However, we have pointed out that if one were to consider the coherence between the concentrations of oxy-hemoglobin (*O*) and deoxyhemoglobin (*D*) as measured with NIRS, care must be taken to properly take into account the intrinsic correlation introduced by the linear relationship between optical intensities and *O* and *D*. In general, the existence of an intrinsic correlation between two signals results in a greater critical value of significance for their coherence.

We also propose to use a wavelet-transform-based (or STFT-based) measure of coherence, which allows for a time-resolved assessment of significant coherence during the course of a CHS measurement. This latter result is particularly important because it enables the identification of coherent hemodynamics during periods of induced physiological perturbations as well as during rest periods, in which only spontaneous hemodynamic oscillations are present. The ability to apply CHS to resting subjects, without a need to induce controlled perturbations in ABP, may result in a broader applicability of CHS for monitoring microvascular reactivity, cerebral perfusion, and vascular brain health.

## Figures and Tables

**Table 1.** Summary of the results reported in Figs. 3–6 about the significance of the four different covariation metrics with five methods to generate surrogate data.

		Subject 1	Subject 2	Subject3	Subject 4
$\langle {\mathrm{Coh}}_{T,\mathrm{abp}}^{\mathrm{FT}}\rangle$	GRN	5	3	5	5
	IID1	5	3	5	5
	IID2	5	0	3	5
	FT1	5	2	5	5
	AAFT1	5	2	5	5
$\langle {\mathrm{Coh}}_{T,\mathrm{abp}}^{\mathrm{WT}}\rangle$	GRN	5	3	4	5
	IID1	5	3	4	5
	IID2	5	2	3	4
	FT1	5	3	4	5
	AAFT1	5	3	4	5
$\langle {\mathrm{PSI}}_{T,\mathrm{abp}}^{\mathrm{HT}}\rangle$	GRN	5	1	3	5
	IID1	5	1	3	4
	IID2	5	1	2	1
	FT1	5	1	2	3
	AAFT1	5	1	2	3
$\langle {\mathrm{PSI}}_{T,\mathrm{abp}}^{\mathrm{WT}}\rangle$	GRN	5	3	4	5
	IID1	5	3	4	5
	IID2	4	3	2	3
	FT1	4	3	4	5
	AAFT1	4	3	4	5

*Note*: Each entry is an integer in the range [0, 5] which corresponds to the number of induced frequencies that were significant according to a particular metric and surrogate data.

**Table 2.** Number of frequencies (out of the five considered frequencies of 0.045, 0.056, 0.0625, 0.071, and 0.023 Hz) that feature significant coherence or phase synchronization during cyclic thigh cuff occlusions and at baseline, for the three non-stationary metrics and thresholds obtained with the surrogate data method FT1.

		Subject 1	Subject 2	Subject 3	Subject 4
$\langle {\mathrm{Coh}}_{T,\mathrm{abp}}^{\mathrm{WT}}\rangle$	Oscillations	5	3	4	5
	Baseline	0	0	5	0
$\langle {\mathrm{PSI}}_{T,\mathrm{abp}}^{\mathrm{HT}}\rangle$	Oscillations	5	1	2	3
	Baseline	0	0	0	0
$\langle {\mathrm{PSI}}_{T,\mathrm{abp}}^{\mathrm{WT}}\rangle$	Oscillations	4	3	4	5
	Baseline	0	0	3	1
